# Neural Correlates of Sound Localization in Complex Acoustic Environments

**DOI:** 10.1371/journal.pone.0064259

**Published:** 2013-05-14

**Authors:** Ida C. Zündorf, Jörg Lewald, Hans-Otto Karnath

**Affiliations:** 1 Division of Neuropsychology, Center of Neurology, Hertie Institute for Clinical Brain Research, University of Tübingen, Tübingen, Germany; 2 Department of Cognitive Psychology, Ruhr University Bochum, Bochum, Germany; 3 Leibniz Research Centre for Working Environment and Human Factors, Dortmund, Germany; 4 Department of Psychology, University of South Carolina, Columbia, South Carolina, United States of America; UNLV, United States of America

## Abstract

Listening to and understanding people in a “cocktail-party situation” is a remarkable feature of the human auditory system. Here we investigated the neural correlates of the ability to localize a particular sound among others in an acoustically cluttered environment with healthy subjects. In a sound localization task, five different natural sounds were presented from five virtual spatial locations during functional magnetic resonance imaging (fMRI). Activity related to auditory stream segregation was revealed in posterior superior temporal gyrus bilaterally, anterior insula, supplementary motor area, and frontoparietal network. Moreover, the results indicated critical roles of left planum temporale in extracting the sound of interest among acoustical distracters and the precuneus in orienting spatial attention to the target sound. We hypothesized that the left-sided lateralization of the planum temporale activation is related to the higher specialization of the left hemisphere for analysis of spectrotemporal sound features. Furthermore, the precuneus − a brain area known to be involved in the computation of spatial coordinates across diverse frames of reference for reaching to objects − seems to be also a crucial area for accurately determining locations of auditory targets in an acoustically complex scene of multiple sound sources. The precuneus thus may not only be involved in visuo-motor processes, but may also subserve related functions in the auditory modality.

## Introduction

Localizing sounds in a complex acoustic environment is a frequent and impressive challenge of every day behavior. In general, our capacity to detect and selectively attend to one particular sound source in a noisy environment is remarkable. This daily experience – also referred to as the *cocktail party phenomenon* [1] – represents an enormous challenge to neural processing in the auditory system, since extraction and localization of the stimulus of interest among others requires simultaneous analysis of several acoustic features, such as pitch, timbre, and spatial cues.

Electrophysiological recording and anatomical tracing studies in primates [2], [3], as well as functional imaging studies in humans [4]–[8] have suggested an auditory dual-pathway model, assuming that auditory spatial and nonspatial information are processed in specialized pathways, namely a posterodorsal stream primarily processing information on sound location and an anteroventral auditory stream preferentially processing non-spatial information on spectrotemporal characteristics of sound (for review, see [9], [10]). A meta-analysis of 36 imaging studies addressing spatial and nonspatial auditory tasks has revealed further support for this assumption [11]. While the planum temporale (PT) was involved in both spatial and non-spatial aspects in the majority of studies, the inferior parietal lobule (IPL) and areas around the superior frontal sulcus (SFS) were more frequently involved in the processing of spatial, than non-spatial auditory aspects. Likewise, the inferior frontal gyrus (IFG) and anterior regions of the temporal lobe were more frequently found to be involved in non-spatial, than spatial, auditory processing although there is direct experimental evidence demonstrating that these regions play a significant role in spatial analysis [12]–[15]. Further studies reported a dissociation of more posterior spatial and more anterior non-spatial processing within temporal lobe [16], [17].

The auditory tasks previously used to identify brain regions involved in spatial analysis have in common that they implemented sound locations presented in isolation. Several imaging and electrophysiological studies with humans and animals have focused on the neural correlates of auditory scene analysis, i.e., the process by which the auditory system separates sounds of interest from competing sound sources, as in the “cocktail party situation” (for review, see [18], [19]). Many of these studies implemented a classical paradigm for auditory stream segregation [20]. In this approach, sequences of two alternating auditory stimuli vary in any acoustic feature (pitch, fundamental frequency, timbre, interaural time difference, or presentation rate) and thus are perceived as either one or two discrete sound streams. Electrophysiological studies found an increased response to the second tone as a function of frequency separation [21], an enhancement of the auditory evoked response in fronto-central scalp regions related to streaming build-up, and a right hemisphere dominance for segregation [22]. Intracranial EEG (iEEG) data on patients with epilepsy demonstrated the involvement of the inferior and middle frontal gyri, as well as the posterior part of the superior temporal gyrus (STG) and perirolandic cortex in auditory streaming [23]. On the other hand, results from neuroimaging studies seem to be inconsistent. Gutschalk and colleagues [24]–[26], focusing on auditory cortex, found that Heschl’s gyrus (HG), planum temporale (PT), and anterior areas of auditory cortex bilaterally play an important role in the separation of auditory streams. Unlike that, Deike et al. [27], [28] suggested that the left auditory cortex is specifically concerned with auditory stream segregation. Moreover, whole-brain imaging studies argued in favour of an involvement of intraparietal sulcus (IPS) [29] and thalamo-cortical loop in sound segregation [30].

In contrast to the abovementioned studies that used tasks with sequences of isolated sounds, further experiments have implemented simultaneous presentation of auditory stimuli. Many of these studies focused on speech intelligibility with multiple speakers. The results suggested involvement of several regions beyond auditory cortex and STG, namely the IFG in listening to dichotically displayed sentences [31], the supplementary motor area (SMA), medial frontal, precentral, and supramarginal gyri in listening to two superimposed stories [32], and the frontoparietal attention network in listening to dichotically presented syllables or sounds [33]. Alain et al. [34], [35] proposed a left lateralized thalamo-cortical network for segregation of superimposed vowels. These authors reported a negative wave superimposed on the N1 and P2 deflections of the auditory evoked potential, which may reflect processes of auditory streaming. Hill and Miller [36] showed that directing attention to one particular talker in a “cocktail party situation” involved IFG, dorsal prefrontal cortex, superior parietal lobule (SPL), and IPL relative to rest, whereas selecting a target among others, based either on pitch or location, was correlated with activation in bilateral posterior STG and superior temporal sulcus (but not HG) as well as in insula, frontoparietal cortex, basal ganglia, and cerebellum. A further approach [37] used consonant or dissonant superimposed harmonic complexes, evoking the percept of one or two streams, respectively. Multiunit recordings in monkeys and iEEG in humans demonstrated oscillatory activity in HG when dissonant chords were displayed, but little or no oscillations for consonant chords [37]. Mistuned harmonics have also been used to induce a pop-out effect of an auditory object from the overall auditory stimulus. The electrophysiological correlate of such an effect has been termed object-related negativity (ORN), which is characterized by a biphasic wave peaking 150–350 ms after sound onset [38], [39]. Moreover, IPS activity was observed using similar abstract stimulation, which may be related to a figure-ground auditory segregation [40].

Despite these previous approaches to the problem of hearing in the “cocktail party situation”, the neural mechanisms underlying sound localization in a complex acoustic environment have remained unclear. The present study aimed to reveal the neural correlates of active localization of an auditory target object when several sounds were presented simultaneously at different positions, thus simulating a real-life “cocktail party situation”. As localizing sounds in a complex acoustic environment involves the simultaneous processing of non-spatial and spatial acoustic features, we predicted activity in an extensive network including auditory cortex and frontoparietal regions. This expected complexity of activation patterns necessitated the disentanglement of the differential aspects of neural analysis using various contrasts. For this purpose, we contrasted the main localization task with passive listening of the same complex auditory scene and with localizing individually presented sounds, thus highlighting the processes underlying active efforts in sound localization in a complex acoustic environment and target sound segregation from the competing distracters, respectively. In addition, the main localization task was contrasted with a task in which subjects had to determine the number of sounds presented in a sequence. The rationale for this contrast was to elucidate the spatial aspects implicated in the “cocktail” task while accounting for its attentional demands. Because of their high ecological validity and spectral richness natural environmental sounds were used.

## Materials and Methods

### Subjects

Twenty healthy subjects (ten females; age range 20–36 years; mean age 27.3, SD ±4.1) participated in the study. All subjects were right-handed as revealed by self report. Participants gave their written informed consent; experiments were carried out following the ethical standards laid down in the 1964 Declaration of Helsinki. The study was approved by the Ethics Committee of the University of Tübingen, Germany. Prior to experimentation, standard audiometry was obtained from each subject. All subjects included in the study had hearing thresholds up to 20 dB HL (hearing level) for the following frequencies: 0.25, 0.5, 1, 1.5, 2, 3, 4, 6, and 8 kHz.

### Experimental Conditions

The main experiment comprised four conditions:

1. Localization of a single target sound (*“single”* condition): One of five possible target sounds was presented in isolation at one of five possible virtual locations. Subjects were instructed to localize the target sound (see below).
2. Localization of a target sound in a “cocktail party situation” (*“cocktail”* condition): Five different sounds were presented simultaneously, each from a different direction. Twenty different auditory scenes were created by different combinations of the five sounds and the five virtual locations. Subjects were instructed to localize the target sound (see below).
3. Passive listening (*“passive”* condition): subjects heard the same auditory scenes as in the *“cocktail”* condition. However, no target sound was introduced. The subjects were instructed not to pay attention to any particular sound and to relax (see below).
4. Sound sequence (*“sequence”* condition): One to 5 sounds were presented consecutively from the same diotic position (see below). Twenty different sound sequences were designed; four with each possible number of sounds; i.e., 1, 2, 3, 4, or 5 sounds were presented within a sequence. Subjects were instructed to carefully listen and report the total number of different sounds presented in the sequence afterwards (see below). Because both the number of sounds in each sequence and the duration of each segment were unpredictable for the subject, sustained attention during the entire stimulus presentation was necessary to complete successfully the counting task.

### Stimuli

Auditory stimuli consisted of five different natural environmental sounds (dog barking; baby crying; telephone ringing; man laughing; cuckoo clock), taken from an online sound library [41]. The sounds were selected based on their familiarity and recognizability.

**Figure 1:**
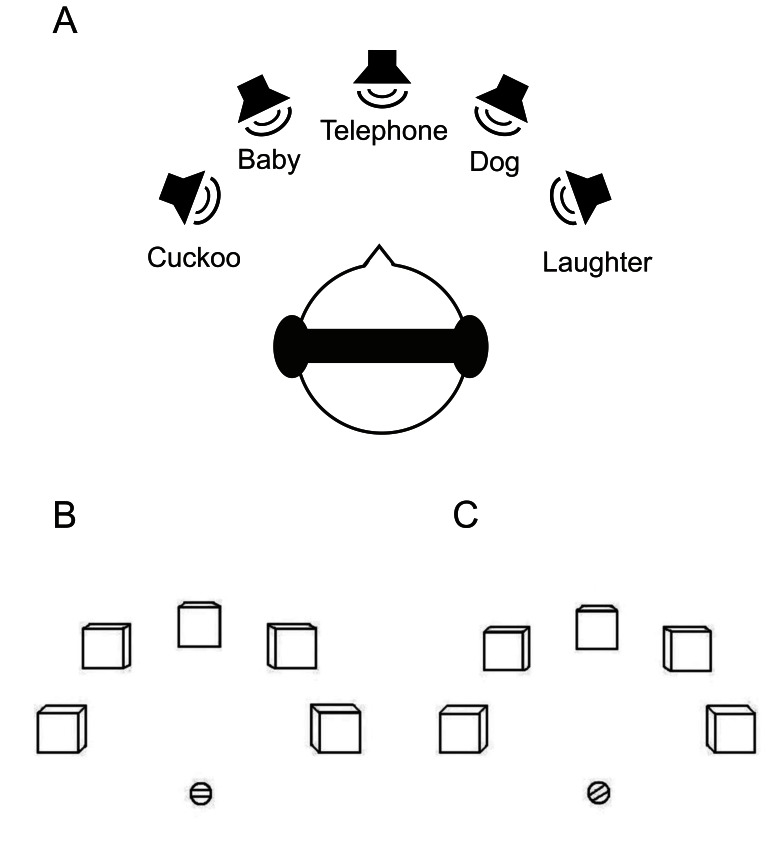
Auditory and visual stimuli. (**A**) Example of one virtual auditory scene used for the *“cocktail”* and *“passive”* conditions. Each sound was presented as coming from a different location. (**B**, **C**) Visual stimuli. Each box represented a sound source (*“single” and “cocktail”* conditions) or from left to right the total number of sounds presented in a sequence of sounds (*“sequence”* condition). Subjects were instructed to perform a saccade as the response in each trial. In the “active” tasks (i.e., *“single”, “cocktail”,* and *“sequence”* conditions), the slot in the circle, which served as saccade starting point, was presented horizontally (neutral) hence not cueing any particular direction. (**B**). In the *“passive”* condition, the saccade direction was cued by the slot in the circle (**C**).

In the *“single”*, *“cocktail”*, and *“passive”* conditions, all sounds were presented for 2 s at identical total sound power (root-mean-square amplitude). If the original sound was longer than 2 s, excessive parts were cut out. In case it was shorter, a segment of the required length of the original sound was copied and appended without acoustic transients between segments. Both pitch and harmonics-to-noise ratio (HNR) were relatively similar for all stimuli as observed using the software Praat (www.fon.hum.uva.nl/praat/; [42]). Spectrograms as well as a detailed description of the sounds are given in Zündorf et al. [43]. In order to present virtual sound locations via headphones, sound files were convolved with generic head related transfer function (HRTF) filters [44]. As described previously [45], each sound was passed through HRTF filters delivered by Tucker Davis Technologies (Alachua, FL, USA), using the RPvds graphical design tool software in combination with a TDT RP2.1 real-time processor system. The HRTF filter coefficients were derived from a set of measurements conducted with a Knowles Electronic Mannequin for Acoustic Research (KEMAR) under anechoic conditions [36]. The HRTFs were recorded at 50 kHz using a KEMAR with head size of 14 cm (from ear to ear), 20.3 cm (from back-of head to tip-of-nose), and 22.9 cm (from top-of-head to tip-of-chin) and with the original KEMAR small sized pinnae [46]. Each HRTF was stored as a 256-tap FIR filter. For each sound, virtual locations were implemented at five different azimuth positions at 0° elevation, either from straight ahead (0°), or from 22.5° or 45° to the left or right (see Fig. 1A). Each sound file for simultaneous presentation of five virtual locations was created by digitally mixing the five different waveforms, with each of the five original sounds located at a different virtual location. Localization of the five virtual locations used in the single and cocktail conditions was assessed prior to the main experiment in the same participants. Subjects were asked to listen to the sounds presented via headphones (K 271 STUDIO, AKG, Austria) and adjust a hand pointer towards the virtual location of the target sound in the azimuthal plane, employing the same response method and experimental set-up as described in detail in Zündorf et al. [43]. This test consisted of 100 trials, 50 trials for the “single” condition and 50 trials for the “cocktail” condition. All sound stimuli used in the main experiment were presented as targets, with 10 repetitions. The resulting mean pointing directions (± SD) for each virtual target location were: virtual target −45°, response −65.57° ±12.00; virtual target −22.5°: response −56.08° ±16.62; virtual target 0°: response −0.33° ±9.32; virtual target 22.5°: response 48.46° ±11.46; virtual target 45°: response 68.48° ±10.59. Although the eccentricity of perceived locations was consistently overestimated (as is known to usually occur with stimulation via headphones; cf., e.g., [47]), the results of this measurements indicated that subjects could clearly distinguish between the five target locations. This was statistically confirmed by the outcome of a repeated-measures ANOVA computed for the five positions (*F*_(1.54, 29.43)_ = 443.85, *p*<0.001, Greenhouse-Geisser corrected), with all pairwise post-hoc comparisons yielding significant results (*p*<0.001).

In the *“sequence”* condition, segments of the original sounds were presented consecutively, thus forming sequences of different sounds. Depending on the overall number of sound segments contained in the sequence (one, two, three, four, or five) the duration of each sound segment was adapted to 400, 500, 600, 800, 1000, 1400, or 2000 ms. Sections of the specified length were cut out from the original sound in a way that the stimulus was still recognizable, as was confirmed during practice trials. In the *“sequence”* condition, sounds were displayed diotically, resulting in a centrally located intracranial percept, in order to minimise spatial cues available to the subject.

All sound files were saved at 44.1 kHz sampling rate and 16-bit resolution. Sound duration and sound power were adjusted using the software Cool Edit 2000 (Syntrillium Software Corporation, Phoenix, AZ, USA). Stimuli were converted to analog form via a PC-controlled, 16-bit soundcard (Audigy 2NX, Creative Labs, Singapore) and were presented at a sound pressure level of approximately 70 dB(A) via MR-compatible headphones (Optime 1, MR confon GmbH, Magdeburg, Germany).

### Behavioral Responses

In all four conditions, subjects were instructed to respond to stimuli via saccadic eye movements after sound offset. This method was chosen since eye movements are normal responses to sound sources in everyday life. The visual stimuli for the saccades consisted of five boxes, designed to represent the five auditory stimuli (Fig 1B, C). In the *“single”* and *“cocktail”* conditions, the positions of the boxes denoted the five auditory positions in space; in experimental condition 4 (“sequence”), they represented from left to right the amount of different sounds displayed in a row (*n* = 1 to *n* = 5). In these three conditions, the circle below the boxes showed a horizontal (neutral) slot throughout (Fig. 1B). In experimental condition 3 (*“passive”*), the subjects were not supposed to attend to any particular sound, but saccadic responses were required; thus the slot in the circle was randomly directed towards one of the five possible boxes (Fig. 1C), indicating the saccade direction to one of the five boxes after every sound. An LCD (800×600 pixels, refresh rate 60 Hz) projector was used to project the visual stimuli onto a screen. Subjects viewed the projection via a mirror positioned on the head coil of the MRI scanner. Eye movements were recorded throughout the whole experiment using an eye-tracking system (SensoMotoric Instruments, Teltow, Germany) at a sampling rate of 50 Hz.

For analysis of the behavioural data, saccade end points of each trial were extracted and compared with the auditory target locations. Saccadic responses were classified as correct if the subject’s saccade end point was within the box representing the actual target sound; otherwise the subject’s response was categorized as incorrect. The total number of correct responses was used as a measure of the subject’s performance. All subjects were trained prior to experimentation; in addition to accomplishing the “single” and cocktail” tasks using a hand pointer (see *Stimuli*), each participant underwent a complete experimental run before the scanning session. All recruited subjects were able to adequately accomplish the task.

### Procedure

**Figure 2:**
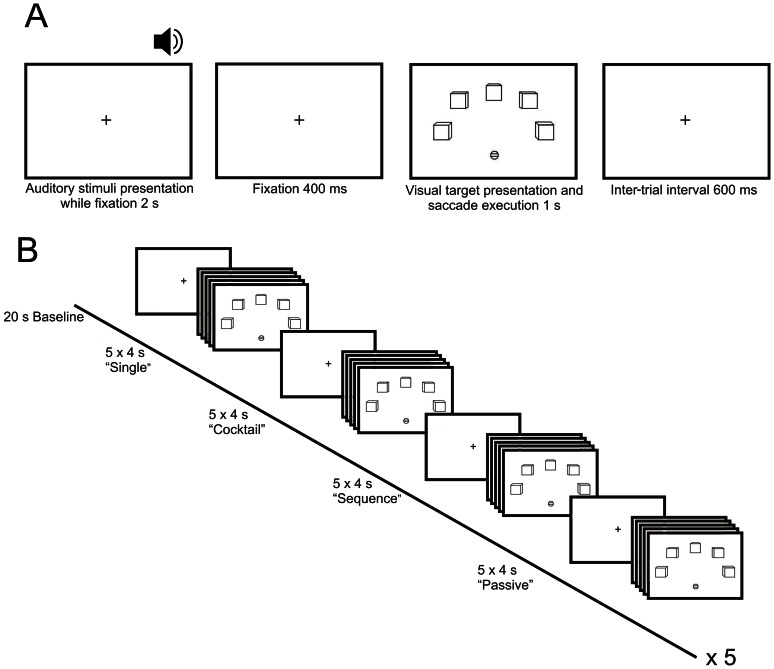
Experimental procedure. (**A**) Trial structure. Each trial began with the presentation of the auditory stimulus while the subject fixated at the fixation cross for 2 s. The auditory stimulus was followed by a 400 ms interstimulus interval and, subsequently, by the presentation of the visual saccadic-response stimulus for 1 s. The intertrial-interval lasted 600 ms. (**B**) Experimental run. Each run comprised 5 blocks of each condition, and each block consisted of a sequence of 5 trials. Between blocks, a 20-s rest period was inserted. Conditions were presented in a fixed order: “*single” – “cocktail” – “sequence” – “passive”*.

In all experimental conditions, each trial began with the auditory stimulus (2 s duration) and a subsequent 400 ms interstimulus interval, which was followed by the saccadic response stimulus (1 s duration). An inter-trial interval of 600 ms was implemented, thus resulting in a trial rate of one per 4 s (Fig. 2A). The order of sound stimuli within blocks was pseudo-randomized in that successive repetitions of identical or similar auditory scenes or sound arrays did not occur. All subjects completed 4 runs. Each run comprised 5 blocks of each experimental condition, and each of these blocks consisted of a sequence of 5 trials, all corresponding to the same condition. In the *single* and *cocktail* conditions, subjects were instructed to attend to the same target sound (Fig. 2B). A baseline, consisting of central fixation to the fixation cross, was implemented between blocks. Each block/baseline period lasted 20 s. The blocks were presented in the following order of conditions: *“single” – “cocktail” – “sequence”* – *“passive”*.

Although a fixed succession of blocks is not optimal for imaging purposes due to the lack of counterbalance among conditions and hence the control for the order of effects, this approach was chosen for the following reasons. To avoid further task instructions and its potential confounds, the target stimulus presented in the initial *“single”* condition always served as the cue for the subsequent *“cocktail”* condition. That is, in the *“cocktail”* condition, the subjects were instructed to keep track of the sound that was previously presented in isolation. Moreover, the preceding individual presentation of the sound may facilitate the segregation of the target sound from the auditory scene in the subsequent “*cocktail”* condition. The *“sequence”* condition, in which subjects had to report the total number of sounds, preceded the passive listening task. This order was adequate since the subjects were not supposed to focus on any specific sound during the *“passive”* condition, and the *“sequence”* condition may have prevented any attentional priming of a particular sound. If the “*passive”* condition would have been presented immediately after the “*cocktail”* or the “*single”* tasks, the subject might not have been able to ignore that sound stimulus that had served as a target in the preceding condition, thus counteracting genuine unattended hearing, as was the aim with the “*passive”* condition.

### Functional Data Acquisition and Analysis

The experiment was conducted using a 3-T whole-body MRI scanner (Magnetom Trio; Siemens, Erlangen, Germany) with a 12-channel head-coil system. T2*-weighted echo-planar images were acquired in transversal orientation covering the whole brain (TR = 2.5s; TE = 40 ms; flip angle 90°; FOV = 192×192 mm; 64×64 matrix; 33 interleaved acquired slices, slice thickness 3 mm, slice gap 0.3 mm) for BOLD-based imaging. Additionally, high resolution T1-weighted anatomical volumes were acquired using an MP-RAGE sequence (TR = 2.3 s; TE = 2.92 ms; flip angle 8°; FOV = 256×256 mm; 256×256 matrix, 176 sagittal slices, slice thickness 1 mm).

Data were preprocessed and analysed using Statistical Parametric Mapping (SPM8, Wellcome Department of Imaging Neuroscience, London, UK) implemented in MatLab 7.5 (TheMathWorks Inc., Natick, MA, USA). The first four images of each run were discarded to allow the MRI signal to reach the steady state. The remaining scans were realigned to the first image to correct for head movements, coregistered to the subjects’ T1 volumes, and normalized to the MNI space applying the unified segmentation normalization procedure [48]. Finally, images were smoothed using a 8-mm full-width half-maximum Gaussian kernel.

The first level analysis included a removal of low-signal frequency drifts using a high-pass filter of 300 s [49]. Each trial was convolved with a canonical hemodynamic response function, as implemented in SPM8. Besides the four experimental conditions, six additional covariates to capture residual movement-related artefacts (three rigid-body translations and three rotations) determined from the realignment procedure were included in the design matrix. Although the tasks were implemented in blocks of five trials throughout the experiment, each single trial was analysed as a separate event. Specific effects of the experimental conditions were tested using one-sample *t*-tests. For further group level analysis, a one-sample *t*-test for the contrast *“cocktail”*>baseline and a one-way ANOVA within subjects (as implemented in SPM8) based on the individual contrast of each condition were computed. Two additional group analyses were computed to rule out potential confounds in the data, namely *(1)* inclusion of the subjects' performance as a covariate and *(2)* modelling the first trial of each block separately to account for cueing effects. All activations reported survived a threshold of *p*≤0.05, FWE corrected (unless otherwise stated). Activations were projected onto the standard single-subject MNI brain template “Colin27”. All coordinates refer to the MNI space.

## Results

### Behavioural Results

Saccadic performance was almost perfect in the *“passive”* condition, in which the direction of the slot in the circle of the visual response stimulus indicated the saccade direction to one of the five boxes (mean percentage of correct responses across subjects: 96.8% ±3.2 SD). Similarly, subjects performed fairly well in the “*sequence*” condition (mean 93.0% ±4.4 SD). As expected from the higher task difficulty associated with active localization, performances in the *“single”* and *“cocktail” conditions* were lower than in the two other conditions, even though still sufficient (*“single”*: mean 85.7% ±5.5 SD; *“cocktail”*: mean 74.8% ±7.7 SD). In all conditions, subjects performed far above chance level (20.0%). A repeated-measures one-factor ANOVA showed significant differences in performances between conditions (*F*_(1.93,36.64)_ = 95.68, *p*<0.001, Greenhouse-Geisser corrected). Pairwise comparisons revealed significant differences between all conditions (*p*<0.001, Bonferroni corrected for multiple comparisons).

### Imaging Results

**Figure 3:**
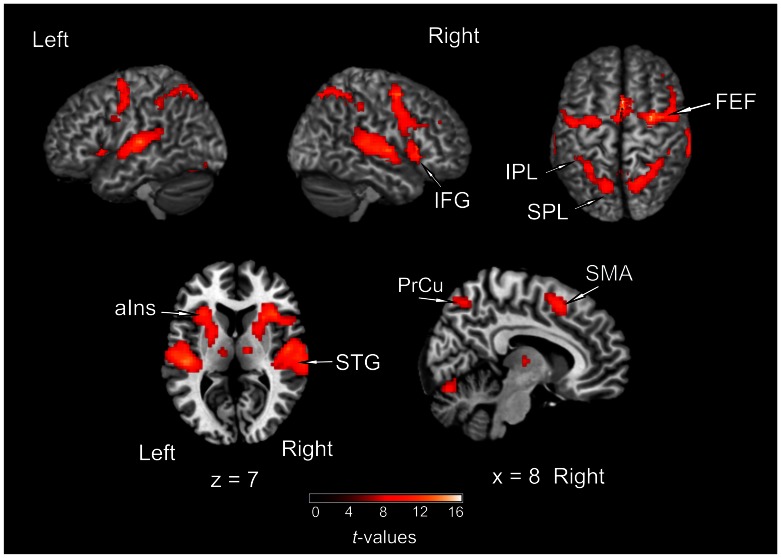
Activations of brain regions as revealed by the contrast of *“cocktail”* condition versus rest (*p*_FWE_ <0.05). STG, superior temporal gyrus; FEF, frontal eye fields; IPL, inferior parietal lobule; SPL, superior parietal lobule; IFG, inferior frontal gyrus; PrCu, precuneus), aIns, anterior insula; SMA, supplementary motor area. The color code refers to *t*-values.

To investigate activations related to the active localization of sounds when multiple competing sound sources were present, we firstly contrasted the *“cocktail”* condition to baseline (rest). Activity was observed along auditory cortex, including posterior STG, HG, and PT, all bilaterally. A further cluster covered the putamen and extended to the anterior insula, including parts of the IFG. Further activity was observed in parietal lobe (comprising SPL, IPL, IPS, and precuneus) as well as in FEF, SMA, and thalamus, all bilaterally (Fig. 3; Table 1).

**Table 1:** Regions with significant activation for each contrast, main analysis.

Region	MNI coordinates*x y z*	*z*-value	Cluster Size [Voxels]	*p*-value
*“Cocktail”*>baseline				
Right FEF	30 −3 54	7.14	740	<0.0001
Right SMA	3 15 51	7.12	194	<0.0001
Right STG	51 −9 3	6.86	620	<0.0001
Left anterior Insula	−30 24 3	6.78	237	<0.0001
Left STG	−60 −21 9	6.77	509	<0.0001
Left IPL	−30 −51 48	6.29	242	0.003
Right SPL	15 −63 51	6.28	337	0.003
Left FEF	−27 −3 51	5.97	248	0.009
*“Cocktail”*>*”passive”*				
Right STG	63 −33 12	6.98	532	<0.0001
Left STG	−66 24 12	6.54	292	<0.0001
Left SMA	−6 18 45	6.33	106	<0.0001
Left anterior Insula	−33 27 0	5.77	78	<0.0001
Left IFG	−42 9 27	5.33	49	<0.0001
Right FEF	48 3 48	5.07	11	<0.0001
*“Cocktail”*>*“single”*				
Left STG	−57 −30 9	7.28	204	<0.0001
Right STG	57 −18 6	5.31	11	<0.0001
Left IFG	−39 12 27	4.94	12	0.012
*“Cocktail”*>*“sequence”*				
Right Precuneus	6 −51 54	6.08	66	<0.0001
Left PT	−48 −27 6	4.87	4	<0.0001

Local maxima employing a voxel-level threshold of *p*≤0.05, FWE-corrected for multiple comparisons and cluster size of more than 10 voxels (except for the “cocktail”>“sequence” comparison). FEF, frontal eye fields; SMA, supplementary motor area; STG, superior temporal gyrus; IPL, inferior parietal lobule; SPL, superior parietal lobule; IFG, inferior frontal gyrus.

**Figure 4:**
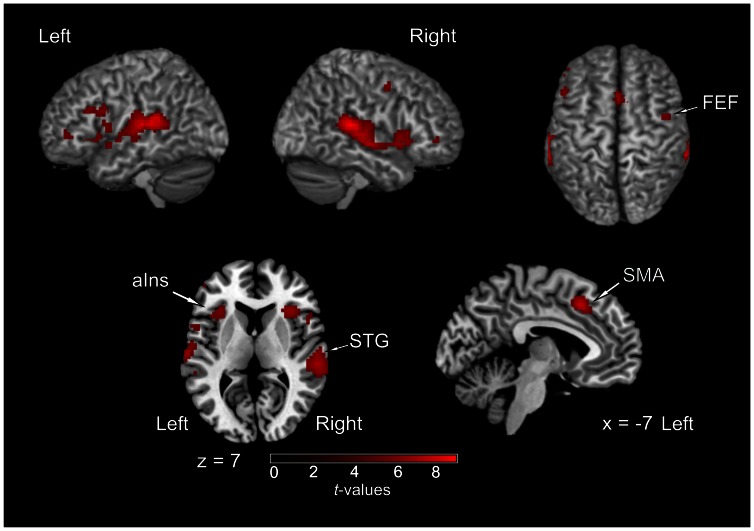
Activations of brain regions as revealed by the contrast of *“cocktail”*>*“passive”* (*p*_FWE_ <0.05). STG, superior temporal gyrus; IFG, inferior frontal cortex; FEF, frontal eye field; SMA, supplementary motor area.

**Figure 5:**
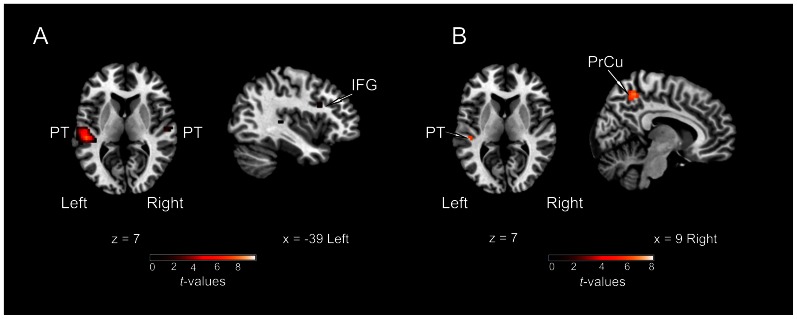
Areas related to the localization of sounds in a “cocktail-party situation”. (**A**) Activations of brain regions as revealed by the contrast of *“cocktail”*>*“single”* (*p*_FWE_ <0.05). The contrast resulted in a major activation of auditory cortex, specifically in the planum temporale (PT). Two further small clusters in right PT and left inferior frontal gyrus (IFG) were observed. (**B**) Activation for the contrast of *“cocktail”*>*“sequence”* (*p*_FWE_ <0.05). The only areas active with this contrast were the precuneus (PrCu) bilaterally and a small cluster in left PT.

Two main contrasts between conditions were computed in order to identify areas specifically involved in solving the “cocktail party problem”: *(1)* “*cocktail”*>*“passive”* and *(2)* “*cocktail*”>*“single”*. The contrast of *“cocktail*”>*“passive*” was intended to reveal areas involved in active efforts in sound localization in a complex auditory scene. While providing identical auditory information, the *“cocktail*” and *“passive*” tasks critically differed by the amount of attention required from the subject. Thus, activation revealed by this contrast may reflect the attentional load needed for the segregation and localization of the target sound in the *“cocktail*” task, rather than the preattentive sensory processes of spatial and non-spatial auditory analysis, which may be similar in the *“cocktail*” and *“passive*” conditions. Eye-movement related activation was controlled: In the *passive* condition, subjects were cued to perform a saccade to one of the five possible targets in each trial. Related visuo-motor processes might have been active during this task. Activations were observed along posterior STG, including PT bilaterally (but not HG), anterior insula bilaterally, SMA bilaterally, and right FEF (Fig. 4; Table 1). When applying a more liberal threshold (*p*<0.001, uncorrected), additional activation in the left FEF, IPL bilaterally, and precuneus bilaterally was observed (not shown). The contrast of *“cocktail”*>*“single”* was computed in order to identify areas more specifically involved in extracting the target sound from the complex auditory scene, as both tasks required active localization of sound and differed in the presence of auditory distracters. These conditions did not differ in acoustic power (one virtual sound source compared to five simultaneous virtual sources). Even though the *“cocktail”* task required a higher demand for target detection and identification than the *“single”* task, we hypothesized that this contrast may reveal brain areas recruited to segregate different auditory streams and to select the one of interest, as it occurs in an everyday-life “cocktail party situation”. The results showed a strong left lateralized activation in auditory cortex, specifically in PT. Minor activation clusters were observed in right PT and left IFG (Fig. 5A; Table 1).

The contrast of *“cocktail”*>*“sequence”* was computed to reveal brain areas specifically concerned with the spatial aspect involved in the *“cocktail”* task. In the contrast of *“cocktail”*>*“passive”* (as described above) these processes were elucidated only partially since the activations revealed may reflect the combination of both non-spatial and spatial attentional demands, without any differentiation between these two aspects of auditory analysis. However, both the *“cocktail*” and the *“sequence*” conditions demanded attention to the sounds and comprised the same total sound power (see *Stimuli*) while differing in the spatial aspect: in the *“cocktail”* condition, subjects had to shift their spatial attention towards the location of the target sound, whereas in the “sequence” condition spatial qualities of the sound stimulus were minimised (due to the absence of binaural cues) and were not part of the task. Even though the behavioural results indicated this task to be easier to solve than the “*cocktail”* task, the attentional demand in the “*sequence”* task may have been also relatively high as it required sustained attention during the entire stimulation period (in order to report correctly the count of sounds presented in a sequence). This contrast resulted in a cluster located in the central region of the precuneus bilaterally with peak activation in the right hemisphere, in addition to a small cluster (four voxel) in the left planum temporale (Fig. 5B; Table 1).

A second version of the analyses (contrasts as in the main analyses) was carried out incorporating the performance of the subjects as a covariate, thus accounting for potential effects related to task difficulty in the BOLD signal. The contrast of *“cocktail”*>*“passive”* resulted in only one voxel located in the right posterior STG. The contrast of *“cocktail”*>*“single”* resulted in a single cluster in the left PT, which was smaller in dimension but at the same location as the cluster obtained in the main analysis. The contrast of *“cocktail”*>*“sequence”* did not yield any significant differences (Table 2). These results indicated a high impact of the task difficulty on activation, thus indicating that these activations were specifically related to active efforts necessary for performing localization of sounds in complex acoustic environments.

**Table 2:** Results of analyses *(a)* employing performance as covariate and *(b)* modelling separately the first trial of each block to account for cueing effects.

Region	*MNI coordinates* *x y z*	*z-* value	Cluster size [voxels]	*p-*value
**a. Analyses including performance as covariate**				
*“Cocktail”*>*“passive”* *No significant results*				
*“Cocktail”*>*“single”*				
Left STG	−57 −27 9	5.88	68	<0.0001
*“Cocktail”*>*“sequence”* *No significant results*				
**b. Analysis with separate modelling of the first trial of each block**				
*“Cocktail”*>*“single”*				
Left STG	−51 −27 6	7.53	176	<0.0001
Left IFG	−36 12 24	5.43	20	<0.0001

Local maxima employing a voxel-level threshold of *p*≤0.05, FWE-corrected for multiple comparisons and cluster size of more than 10 voxels. STG, superior temporal gyrus; IFG, inferior frontal gyrus.

A third version of analysis was computed to rule out possible confounds related to the lack of a cue in the *“single”* condition as compared to the *“cocktail”* task, in which the target sound was cued by the preceding *“single”* block. Cueing is known to have significant effects on brain activity insofar as areas related to the cued stimulus might be active during this period while task-irrelevant regions or areas related to distracters might be inhibited (e.g. [50]–[53]). In a blocked presentation of five events, as employed here, a cue might have had a potential effect especially on the first trial of each block, rather than on the remaining trials of the block, when the subjects were well acquainted with (and thus already cued to) the target. Under this assumption, the first trial of each block was modelled separately and the contrast of *“Cocktail”*>*“single”* was computed as in the main analyses. The results differed slightly from the outcome of the main analyses. The clusters in the left PT and left IFG, although less extensive, were also found to be active, whereas activity in right PT, as was obtained in the main analysis, could not be established (Table 2).

## Discussion

Localizing sounds in a cluttered auditory environment is a complex task involving selective attention, auditory stream segregation, sound localization and identification. The present results indicate that this highly demanding task recruits a widespread network involving several cortical areas beyond primary auditory cortex, namely posterior STG, IFG, anterior insula, FEF, SMA, SPL, and IPL. To disentangle the specific contribution of the different brain areas, we contrasted the main experimental task of localizing sounds in a “cocktail party situation” with *(1)* passive listening to elucidate processes underlying active efforts in sound localization in a complex acoustic environment with *(2)* localization of single sounds to highlight the areas involved in target sound segregation from the competing distracters; and with *(3)* hearing of sound sequences to investigate spatial aspects involved in the “*cocktail*” task while accounting for its attentional demands.

The contrast of *“cocktail”*>*“passive”* was implemented to discern brain areas reflecting active efforts during spatial attention on a particular sound source as compared to hearing, but not attending to, a complex auditory scene. The resulting activations were found along the posterior STG, in anterior insula, SMA and right FEF. Activation of further areas that could be expected as part of the typical frontoparietal attention network was not obtained due to the subtraction of eye-movement related activity, which relies on the same network [54]–[58]. As was expected, activation was found in auditory cortex, whereas the HG was not active with the contrast of *“cocktail”*>*“passive”*. Our results support previous findings suggesting that the primary auditory cortex transmits reliably (without attentional modulation) the auditory information to higher-order auditory areas for further processing [36]. The anterior insula, even though not considered to be part of the frontoparietal network, has been found to be active in several studies related to spatial orientation in various sensory modalities [59]–[61].None of these areas were found to be active when the analysis was computed with performance as a covariate. This indicates that the activations obtained with the contrast of *“cocktail”*>*“passive”* in the main analysis may reflect an attentional load-related activity that is task-specific. In other words, this contrast may reflect the active efforts needed to identify, filter out, and localize the target sound in a complex auditory scene.

The second main contrast of *“cocktail”*>*“single”* was computed to identify brain regions involved in separating the sound streams and extracting the one of interest among many distractors, which is a main issue in solving the “cocktail party problem”. The analysis showed that these processes may rely particularly on the left auditory cortex, or more precisely on the left PT. This PT activation was still present when localization performance was included as covariate, thus suggesting that sound segregation may be successful even if the localization of the target sound failed. That is, stream segregation may be necessary, but not sufficient, for accurate determination of target location. As suggested by our results, this latter process may take place in the precuneus. Our present findings may supplement previous observations by Deike et al. [27], [28] who reported left auditory cortex activation for auditory stream segregation with sound sequences. In these studies, either pitch or timbre was varied in two harmonic complexes in order to induce a percept of one or two streams. Similarly, by applying intracranial electro-encephalography, Bidet-Caulet et al. [62] demonstrated specialization of the left auditory cortex in attention selection when concurrent sounds were present, and Alain et al. [35] showed specialization of left auditory areas (HG and PT) in segregation of concurrent vowels. To our knowledge, the present study is the first one showing the involvement of the left PT in filtering out a target sound among many auditory distracters by using a complex and realistic “cocktail party situation”, in which environmental sounds were presented simultaneously from various locations. Interestingly, previous studies have demonstrated right, rather than left, lateralized [22], or bilateral activation for stream segregation [21]. Similarly, Zatorre et al. [8] demonstrated a bilateral activation when subjects listened passively to a mixture of several reversed environmental sounds.

Griffiths and Warren [63] speculated that the PT might be a “computational hub”, processing spectrotemporal patterns associated with the identity of auditory objects as well as spectrotemporal cues related to the spatial location of sound. Our results may support these predictions as well as another previous finding suggesting no selectivity in the PT for auditory object and spatial processing, as was investigated by presenting one or three talkers simultaneously in one or diverse locations or even in motion [64]. In the present study the PT appears to be involved in *(1)* selecting a target sound among distracters by spectrotemporal analysis and *(2)* segregating the sound locations by analysis of the spatial information of each individual sound source. The stronger activity in left, than right, PT during the “cocktail party” task suggests that the left hemisphere, and particularly the PT, is not only specialized for speech functions, but rather for higher-order processing of temporal and spectral sound features. In an evolutionary context, this general specialization of left PT might, however, have been an important prerequisite for the development of the specialization in speech analysis of this area. In fact, it has been shown that left auditory areas process acoustic information with a higher resolution as compared to their homologues in the right hemisphere [65], [66]. However, future studies might clarify the exact contributions of left and right auditory cortices in auditory streaming.

It has to be noted that in the analysis with separate modelling of the first trial of each block (thus accounting for cueing effects) activity was absent in right PT and was reduced in left PT compared with the main analysis. This suggests that the activity revealed by the main analysis may, at least in part, be related to stimulus anticipatory effects, as are typically evoked by cues [50]–[53]. Thus, although the bilateral PT activation found in the main analysis might partially reflect cueing effects, the major activation in left PT (as compared to the main analysis) argues in favour of its pivotal role in sound stream segregation.

We also obtained a small activation cluster in IFG. This area has, on the one hand, been assumed to be part of the “what” auditory network (for review, see [11], as it was shown to be preferentially involved in frequency and pitch processing (e.g. [4], [67]–[70]), auditory working memory [71], sound identification [72], [73], and auditory discrimination under dichotic conditions [33]. On the other hand, an involvement of the IFG in the spatial perception of single sound sources has been demonstrated by positron-emission tomography (PET) [15], fMRI [14], and electrotomography [13], thus indicating a significant role of this region in spatial analysis. It has been hypothesized that the IFG subserves both spatial and non-spatial functions of spectrotemporal analysis and is part of a shared cortical network for *(1)* sound identification by spectrotemporal object-features and *(2)* spatial analysis of realistic sound sources providing spectrotemporal localization cues [13]–[15], [74]. The present results are in alignment with this view insofar as the higher task difficulty with localization of sound in a “cocktail party situation” (as compared to single-sound localization) may primarily refer to a higher demand of spatial and non-spatial spectrotemporal analysis.

Finally, a critical point regarding the contrast of “*cocktail*”>*“single*” pertains to the intelligibility of the sounds. Sounds were doubtlessly more difficult to identify in the *“cocktail”*, than in the *“single*”, tasks. This fact was likely to induce potential confounds in the present data. To account for such effects, further studies using meaningless stimuli, such as noise stimuli, are needed.

The contrast of *“cocktail”*>“*sequence”* was computed in order to investigate the spatial aspects involved in the *“cocktail”* task while accounting for its attentional demands. We assumed that this contrast should have revealed areas specifically concerned with the solution of the spatial aspect of the “cocktail party problem”, that is, the localization of the attended sound among distracter sources. While both *“cocktail”* and *“sequence”* conditions contained the same sounds (with identical sound power) and required attending to the sounds, the most critical difference between these two tasks may have referred to the spatial vs. non-spatial task demands. Nevertheless, it has to be emphasized that conclusions from this contrast have to be treated with caution. The task used in the *“sequence”* condition differed in further important aspects from the *“cocktail”* task. In particular, in the *“sequence”* task sounds were presented sequentially, but simultaneously in the *“cocktail”* task. Thus, the cognitive requirements of both tasks are not directly comparable.

The only activated areas that were obtained by the *“cocktail”*>*“sequence”* contrast were a small cluster in the left PT and a major cluster in the precuneus, which is part of the posteromedial portion of the parietal lobe (for review, see [75]). In line with this finding, nearby activations in superior posterior parietal cortex have been reported in imaging studies on localization of single sound sources (e.g., [4], [8], [11], [13], [14], [60], [76]). Interestingly, an almost identical locus of activation in precuneus as obtained here was found in a PET study by Hugdahl et al. [77] for the contrast of focused attention (to one ear) compared to divided attention (to both ears) in a dichotic listening situation, thus supporting the view that this specific area is a crucial part of the network for auditory spatial attention. Moreover, Mayer et al. [78], [79] found activation in precuneus during endogenous and exogenous auditory re-orienting and interpreted this as a correlate of sound localization processes, when stimuli appeared at unexpected locations. Further studies suggested a role of the precuneus in shifts of attention between spatial locations in the visual and auditory modalities [80], [81] and in auditory spatial and non-spatial shifts of attention [59].

The posterior parietal lobule has been assigned to the posterodorsal auditory pathway, which is assumed to preferentially process spatial auditory information (for review, see [11], [55]). In this more general context, our finding may highlight the crucial role of the posterior parietal cortex in higher-order auditory spatial processing, as had been previously shown by numerous studies using neuroimaging (e.g., [4], [6], [13]–[15], [82]), transcranial magnetic stimulation (e.g., [83], [84]), analyses of brain lesions (e.g., [7], [85]–[88]), and single-neuron studies in non-human primates (e.g. [89], [90]). However, the comparison between the different posterior parietal activation loci found in the present experiment (see Figs. 4, 5B) as well as in previous studies led one to speculate on a potential functional differentiation of inferior and superior aspects of posterior parietal cortex in auditory spatial analysis. The putative homologue of the activation foci found in human IPL with auditory spatial tasks might be the lateral intraparietal region (LIP) of the monkey, which is known to subserve the programming of saccades to visual and auditory targets (for review, see [91]). Thus, as in all contrasts computed here saccade-related activation may have been nullified, it may be not surprising that inferior parietal activation was always absent. On the other hand, further lines of investigation, related to reaching visual targets, have shown involvement of the precuneus in visually guided actions [92], [93]. Lesions in the SPL including the precuneus cause optic ataxia, i.e., gross misreaching of visual targets presented in the periphery of the visual field [94]. Single-neuron recordings in the parietal reach region (PRR) have demonstrated responses related to the programming of reaches to visual and auditory targets (for review, see [91], [95]). Moreover, it has been suggested that the parietal cortex converts the location of auditory events into a system of coordinates available to the visual system for further processing [90]; (for review, see [55]); and further studies have demonstrated that parietal neurons might integrate postural and retinotopic information, thus allowing spatial localization of targets in any modality and in different frames of reference (for review, see [96]). Our results are in alignment with these previous findings insofar as the precuneus may, in addition to its well-known visuospatial/motor functions, play a more general, supramodal role in the computation of coordinates for target-directed motor responses across several frames of reference, accessible for stimuli of any sensory modality [95]. In this way, the precuneus seems to be involved in determining the precise location of relevant stimuli from various sensory modalities as well as in the subject’s preparation to act on it, and may, hence, also be essential for localizing the sound source of interest in a “cocktail party situation”.

The analysis computed with performance as covariate for the contrast *“cocktail”*>*“sequence”* did not reveal any BOLD signal changes, neither in the precuneus nor in other brain areas. Thus, the signal change observed in the precuneus in the main analysis (without performance as covariate) could be interpreted as an attentional load-related activity, reflecting the efforts required to localize the target sound. That is, the neural activity in the precuneus seems to be essential for accurate localization of the target. At the first glance, this conclusion might appear contradictory to the results obtained in the contrast of *“cocktail*”>*“passive*”, as significant BOLD signal changes were lacking in precuneus with this contrast. This could, potentially, be explained by the fact that acoustic stimulation was identical in *“cocktail*” and *“passive*” tasks. Thus, one cannot rule out that during the *“passive”* task signal changes were related to the changes in spatial location of unattended sounds, as was reported by Deouell et al. [97]. Furthermore, it is possible that in the *“passive”* task subjects implicitly attended to specific sounds although they were asked not to do so. However, this seems rather unlikely since in this case the contrast *“cocktail”*>*“passive”* would not have yielded any significant signal changes at all. Also, it is conceivable that during the *“passive”* task some precuneus activity could have been evoked due to the changing orientation of the slot in the circle indicating the direction of the upcoming saccade (see *Behavioral responses*). This latter explanation seems to be likely. As mentioned above, the precuneus is known to process spatial information in multiple sensory modalities, and hence its activity may be modulated by the changes in the visual stimuli that were part of the *“passive”* task in the present study.

Taking into account previous evidence and the present results, the precuneus activity is likely to reflect the neural processes underpinning the localization of the sound source of interest in a complex auditory scene. However, considering the reservations about the contrast of *“cocktail”*>“*sequence”* mentioned above, further studies are needed to finally clarify this issue. The simultaneous presentation of a mixture of different sounds from a unique diotic position combined with a rather content-related task might be a possible way to confirm the role of the precuneus in auditory spatial hearing proposed here.

### Conclusion

In summary, extracting a sound of interest among others recruits preferentially the left PT, while further efforts in localizing the target sound appear to rely on the precuneus. Our data extend previous findings regarding the role of the PT in auditory stream segregation. In fact, this area appears to be involved in active segregation of an auditory object when several sounds are presented simultaneously in different positions, as in a real-life “cocktail party situation”. Also, our results suggest that the precuneus is involved in computing the exact location of sound sources of interest in such an auditory scene. The precuneus thus may not only be involved in visuo-motor processes, but may also subserve related functions in the auditory modality. In conclusion, both the PT and the precuneus seem to be the most essential areas for focussing on a particular sound source of interest in a cluttered auditory environment.
